# Sleep quality is associated with disease severity in adult patients with inflammatory dermatoses

**DOI:** 10.1111/jdv.18505

**Published:** 2022-08-24

**Authors:** Bruno Halioua, Jonathan Taieb, Anne‐Laure Demessant‐Flavigny, Delphine Kerob, Sophie Seité, Caroline Le Floc'h, Charles Taieb

**Affiliations:** ^1^ Dermatologist Paris France; ^2^ Maison de la Dermatologie Société Française des Sciences Humaines de la Peau Paris France; ^3^ APHP, Hôtel Dieu Centre du Sommeil et de la Vigilance Paris France; ^4^ Laboratoire Dermatologique La Roche‐Posay Levallois‐Perret France; ^5^ Patients Priority Department European Market Maintenance Assessment [EMMA] Fontenay Sous‐Bois France

Dear Editor,

Several studies indicate that sleep disturbance (SD) is prevalent in patients with inflammatory dermatoses (IDs).^1^, ^2^ The relationship between sleep quality and disease severity in patients with ID has not been clearly defined. In our study, we aimed to analyse the array of SD caused by ID and to evaluate the interaction between the quality of sleep and ID severity.

A survey was administered to a representative sample of 18–75 years old adult population of five different countries (Brazil, China, France Russia and the USA). All participants answered a web‐based questionnaire on socio‐demographic characteristics and subjective evaluation of sleep quality attributable or not to dermatological disorders. The clinical severity of ID was self‐evaluated [as mild, moderate or severe]). In addition, the patients completed the Dermatology Life Quality Index (DLQI) questionnaire to assess the health‐related quality of life (HRQoL) impairment related to cutaneous symptoms. DLQI scores were divided into 2 groups: DLQI ≥10 (severe impact on HRQoL) and DLQI <10 (slight impact on HRQoL). A chi‐squared test and Fisher's exact test were used to compare differences in categorical data, and an independent *t*‐test was used for continuous variables. Correlations between tested parameters were verified with the Spearman rank correlation test. A value lower than 0.05 was considered statistically significant.

Among the 3857 participants, 2360 were female (61.2%), and 1497 were male (38.8%) (mean ± standard deviation (SD) age 45.0 ± 14.6 years). There were 2313 patients with atopic dermatitis and 1544 with psoriasis. The prevalence of SD was 85.6%. Prevalence of SD is higher in patients with AD than in those with psoriasis (87.5% vs. 82.8%, *p* < 0.05). Patients with SD slept less than subjects without SD (median 6.65 ± 1.49 vs. 7.30 ± 1.12. *p* < 0.05). A total of 10.6% still exclusively reported *sleep*‐*onset insomnia (SOI)* (difficulty in falling asleep), 14.4% reported sleep maintenance insomnia (SMI) exclusively (inability to stay asleep during the night), and 60.8% reported *SOI associated with* SMI. A total of 2.7% reported that SD was consecutive to their ID (SDID), 45% considered that SD was not the consequence of ID (SDID) and 52.3% of both SDID and SDNID. Compared with patients with mild ID, patients with moderate and severe ID were more likely to complain of sleep disturbance (93.3% if moderate and severe vs 81.7% if mild, *p* < 0.05) and slept less (median 6.65 ± 1.49 vs. 7.30 ± 1.12. *p* < 0.05). There was a significant association between the prevalence of SD and severe impact on HRQoL ([95.5% DLQI ≥10 vs. 81.6% if DLQI<10]. *p* < 0.05).

The median nighttime sleep duration was shorter in patients with a severe impact on HRQoL (6.35 vs. 6.90. *p* < 0.05). Moderate and severe ID and severe impact on HRQoL were more prevalent among patients with SOI *with* SMI (Table 1). A significant correlation was noted between DLQI and prevalence of SOI *with* SMI (R: 0.88, *p* < 0.01) (Figure 1).

**Table 1:** Description of the prevalence of sleep‐onset insomnia [SOI] with sleep maintenance insomnia [SMI] according to the severity and impact of the disease [ID].

	DLQI	*n*	%SD	%SOI	%SOI + SMI	%SMI
Mild ID	<10	2127	79.7	12.00	47.90	19.80
	≥10	411	91.7	8.80	71.50	11.40
Moderate and severe ID	<10	616	88.1	10.70	71.60	5.80
	≥10	690	97.8	7.40	84.50	5.90

**Figure 1:**
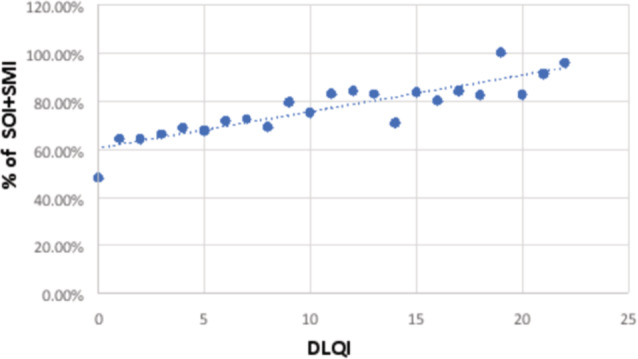
Correlation between DLQI and prevalence of sleep‐onset insomnia [SOI] with sleep maintenance insomnia [SMI].

This is the first epidemiological study evaluating the interaction between sleep quantity and quality and disease severity in adult patients with ID. Our results confirm previous literature suggesting a higher prevalence of sleep disturbance in ID in comparison with 10–20% for the general population.^3^ The reciprocal relationship between SD and ID can be explained by the aetiopathogenic role of proinflammatory cytokines, such as TNF alpha and interleukin‐6, both of which play an important role in the pathogenesis and development of ID.^4^, ^5^ Psychological problems such as depression, anxiety low self‐esteem and fear of being stigmatized can strongly influence sleep quality. Our study showed that DLQI scores were significantly correlated with the prevalence of SOI and SMI. The limit is the absence of comparison of SD in a population without IDs. These findings suggest the importance of early detection and management of SD, which can have a detrimental impact on physical functioning and mental health and may contribute to daytime sleepiness, behavioural problems and depression.^6^, ^7^ It is therefore essential to include questions about SD in the examinations of ID patients.

## Conflict of interest

AL Demessant, D Kerob, S. Seité and C Le Floc’h are employees of La Roche Posay. B Halioua, J Taïeb and C Taïeb declare no conflict of interest.

## Funding information

This project was funded by La Roche Posay.

## Data Availability

The data from the study that generated the published results are available.
